# Is *Pseudomonas aeruginosa* a possible aetiological agent of periodontitis in dogs?

**DOI:** 10.2478/jvetres-2025-0006

**Published:** 2025-02-25

**Authors:** Małgorzata Targońska-Karasek, Izabela Polkowska, Henryk Krukowski

**Affiliations:** Department of Animal Hygiene and Environmental Hazards, Faculty of Animal Sciences and Bioeconomy, University of Life Sciences in Lublin, 20-950 Lublin, Poland; Department and Clinic of Animal Surgery, Faculty of Veterinary Medicine, University of Life Sciences in Lublin, 20-612 Lublin, Poland

**Keywords:** dog, periodontitis, *Pseudomonas aeruginosa*

## Abstract

**Introduction:**

Periodontal diseases are the most frequently diagnosed problem in small animal veterinary medicine. Although their exact cause is not fully understood, bacteria play an important role in their development. *Pseudomonas aeruginosa* is a Gram-negative, rod-shaped, non-spore-forming bacterium. The living environment of this bacterium may be soil and water; however, it can also be found in humans and animals. Antibiotic treatment of periodontitis may be complicated by the carbapenem resistance of some *P. aeruginosa* strains, if these bacteria are found to be an aetiological agent. The aim of the study was to identify all bacterial strains isolated from dog with periodontitis.

**Material and Methods:**

After a clinical examination of a Schnauzer dog in the Department and Clinic of Animal Surgery in the University of Life Sciences in Lublin Faculty of Veterinary Medicine, periodontitis was diagnosed. A swab was taken from the diseased tissue and submitted for microbiological tests. Microorganisms were initially identified by colony morphology, haemolytic pattern and Gram staining, and subsequently by sensitivity tests, VITEK 2 and matrix-assisted laser desorption/ionisation–time-of-flight.

**Results:**

*Pseudomonas aeruginosa* was isolated and identified as a probable aetiological factor of periodontitis in dogs.

**Conclusion:**

In our opinion, attention should be paid to *Pseudomonas aeruginosa* as a possible aetiological factor of periodontal diseases in dogs.

## Introduction

Periodontal disease is one of the most common diseases in adult dogs and affects up to 80% of animals. The aetiology of the disease is poorly studied, although bacteria are known to play a major role (25). Based on the clinical signs, periodontal diseases are usually divided into two groups, gingivitis and periodontitis. The most common clinical signs of periodontal disease include halitosis, ptyalism, altered gingival colour, gingival bleeding, tooth mobility, anorexia and behavioural changes (1, 13). The diagnosis of periodontal diseases in dogs typically involves a clinical examination and dental radiography, which can reveal changes in the structure of the periodontal bone. Microbiological tests, including cultures and bacterial identification, can be useful in determining the specific pathogens present in the oral cavity (23), particularly in light of the highly diverse canine oral flora in healthy as well as diseased animals (25).

*Pseudomonas aeruginosa* is a Gram-negative, rod-shaped, non-spore-forming bacterium with dimensions of 0.5–0.8 μm by 1.5–3.0 μm (5). The living environment of this bacterium may be soil and water; however, it can also be found in humans and animals. It is primarily aerobic but can also grow anaerobically in the presence of NO_3_. Its mobility is provided by the flagellum at the cell’s pole (28). The optimal temperature for the growth of *P. aeruginosa* is 37°C, but it can also grow at temperatures up to 42°C (5). The bacterium does not liquefy gelatin and does not produce indole or β-galactosidase. It is oxidase-positive, lactose-non-fermenting and secretes pigments such as pyocyanin, fluorescein, pyorubin or pyomelanin (18). The bacterium can form complex macrocolonies (biofilms) attached to surfaces or exist free as single-celled organisms (planktonic forms) (28).

In 2000, the entire genome of *P. aeruginosa* was sequenced. It consists of a relatively large circular chromosome (5.5–6.8 Mb) that carries between 5,500 and 6,000 open reading frames, and depending on the strain, sometimes plasmids of various sizes (20).

*Pseudomonas aeruginosa* is an opportunistic bacterium for humans and animals and one of the most important and dangerous microorganisms causing nosocomial infections (8). Infection with *P. aeruginosa* is a particularly important problem for patients with cystic fibrosis, and its treatment is difficult because of the high resistance of this bacterium to antibiotics (8, 15, 24).

The bacterium is naturally resistant to many antibiotics because of the relative impermeability of the cell wall and membrane. Its natural resistance to chemotherapeutic agents of which the spectrum includes Gram-negative bacteria extends to resistance to cotrimoxazole and many β-lactam antibiotics (28). Hospital strains may be resistant to a larger number of drugs, because plasmids containing resistance genes may be transferred between microorganisms by transduction and conjugation. This involves the production of much larger amounts of cephalosporinases in transfer-recipient strains as a result of gene derepression, the production of carbapenemases and the synthesis of membrane pumps moving drugs out of the cell or mutations of proteins responsible for the transport of antibiotics into cells (4, 19). Some strains produce *Pseudomonas*-specific enzymes with a narrow substrate spectrum which degrade some beta-lactam antibiotics (22).

The main aim of the study was to attempt a comprehensive identification of the bacterial strains isolated from a dog with periodontitis. The second aim was the direction of further research attention to *P. aeruginosa* as an aetiological agent of canine periodontitis.

## Material and Methods

### Clinical examination

The owner of a 9-year-old male Schnauzer dog with recurrent oral problems came to the Department and Clinic of Animal Surgery at the University of Life Sciences in Lublin. Periodontal examination and swab collection for microbiological tests were performed on the dog under general anaesthesia before the oral cavity was sanitised. The dental examination included a visual assessment of the gums, the oral vestibule mucosa and the surface of the tooth crowns. Special attention was paid to the occurrence of swelling and redness of the gums, the appearance of ulcers/erosions on the oral mucosa and the presence of bacterial plaque/tartar deposits covering the tooth surfaces. The consistency and tenderness of the gums were assessed by palpation. The measurement of periodontal pocket depth (PPD) was performed using a Williams periodontal probe and was marked in millimetres. The probe was inserted carefully, parallel to the long axis of the tooth. The examination was performed at four measurement points. Pocket depth of 1–3 mm was assessed as gingivitis, depth of 3.1–5 mm as early periodontitis, 5.1–7 mm indicated moderate periodontitis and PPD > 7 mm was a severe periodontitis diagnosis. After a clinical examination, periodontitis was diagnosed. Then, after general anaesthesia, scaling was performed and the tooth crowns were protected with Biochem enamel rebuilding preparation (Chema-Elektromet, Rzeszów, Poland).

### Microbiological examination

A sample was collected using a sterile swab from the periodontal pocket (after rinsing with physiological saline solution). The swab was placed in a collection tube with sterile Amies transport medium (Medlab-Products, Raszyn, Poland) and immediately sent for microbiological analysis. General treatment was implemented based on an antibiotic resistance test and microbiological analysis.

The swab sample was plated as soon as possible on sheep blood agar (Columbia LAB-AGAR + 5% sheep blood; BioMaxima, Lublin, Poland) and was incubated at 37°C for 18–24 h. Microorganisms were initially identified by colony morphology, haemolytic pattern and Gram staining (Color Gram 2 kit; bioMérieux, Marcy-l’Étoile, France).

### Susceptibility test

The antibiotic susceptibility was tested on Mueller–Hinton agar (BioMaxima) using the standard disc diffusion technique (Kirby–Bauer test) as recommended by the Clinical and Laboratory Standards Institute (6). Bacteria were grown on Mueller–Hilton agar at 37°C for 18–24 h. After incubation, a few colonies were reconstituted with sterile physiological saline to a dilution approximating to 0.5 McFarland standards. The bacterial suspension was spread onto a Mueller–Hinton agar plate surface to form a confluent lawn and incubated for approximately 15 min, and afterwards, the agar plates were impregnated with the following antibiotic discs: the aminoglycoside gentamicin at 10 μg; the antipseudomonal cephalosporins ceftazidime and cefepime, both at 30 μg; the antipseudomonal carbapenem imipenem at 30 μg, the antipseudomonal fluoroquinolone ciprofloxacin at 5 μg and the antipseudomonal penicillin piperacillin at 100 μg combined with the β-lactamase inhibitor tazobactam at 10 μg. The antibiotics tested against *P. aeruginosa* were selected according to the recommendations of Magiorakos *et al*. (21). Plates were read after 24 h incubation, and the results were recorded.

### Identification of microorganisms using VITEK 2

The biochemical identification of microorganisms was performed with VITEK 2 (bioMérieux). A bacterial suspension was prepared from the isolated colonies grown in the medium in 3 mL of sterile saline in a clear polystyrene test tube. The turbidity of the suspension was adjusted to a McFarland standard of 0.5 with the help of a VITEK 2 DensiCheck instrument. The time between the preparation of inoculum and filling of the VITEK 2 card was always less than 30 min. Identification with the VITEK 2 compact system was performed according to the manufacturer’s instructions using the Gram-negative (GN) card.

### Identification of microorganisms using MALDI Biotyper

A Microflex LT/SH MALDI Biotyper system (Bruker, Billerica, MA, USA) and IVD library v.12 containing 4,194 species from 2022 (Bruker) was used. The spectra were compared with the library entries with IVD Compass 4.2.100 software (Bruker). The standard identification protocol provided by the manufacturer of the spectrometer was followed, and α-cyano-4-hydroxycinnamic acid was adopted as a matrix.

### DNA analysis

Bacterial isolates were passed several times into the Petri dishes with Mueller–Hinton blood agar to multiply the culture. The Petri plates were cultured for 24 h with agar for DNA isolation, which was carried out from individual bacterial colonies using a Tissue & Bacterial DNA Purification Kit (EurX, Gdańsk, Poland) according to the product protocol. The concentration and quality of the isolated DNA were evaluated using a NanoDrop One spectrophotometer (Thermo Scientific, Wilmington, DE, USA). The obtained DNA samples were diluted with nuclease-free water (EurX) to 20 ng/μL for further analyses.

For molecular identification of bacteria, a multilocus sequence analysis strategy was pursued. Four primer pairs were designed, one amplifying a fragment of the *16S rRNA* sequence, and three doing so for three housekeeping genes: *gyrB*, encoding gyrase subunit B, which is a subunit of an important bacterial enzyme that catalyses the adenosine triphosphate–dependent negative supercoiling of circular double-stranded DNA; *rpoB*, encoding the β-subunit of RNA polymerase; and *rpoD*, encoding sigma factor binding RNA polymerase. The sequences of the primers used in genetic identification are presented in Table 1.

**Table 1.** Primers sequences used in the study

Gene	Forward primer	Reverse primer	Expected product length (bp)
*16srRNA*	AGGGAGCTTGCCTTGGATTC	GGATGCAGTTCCCAGGTTGA	557
*gyrB*	CCGGAGACCTTCAGCAACAT	TTGCCCATCTCCTGTTCCAC	548
*rpoD*	AAAGAGTTGATCGCCCGAGG	TGCTGTCGTCGCTTTCTTCT	583
*rpoB*	TTGTTTCTGTTGCTGCGTCG	GATTTCCTCTGGGCCAAGCT	572

All PCRs were performed in 25 μL volumes containing 20 ng of genomic DNA, 2× PCR Mix Plus Red (A&A Biotechnology, Gdańsk, Poland) and 0.2 μM of each primer. Amplification was carried out in a Biometra TOne thermal cycler (Analytik Jena, Jena, Germany). The cycling conditions of the PCR were as follows: one cycle of 5 min at 95°C; 30 cycles of 30 s at 95°C, 30 s at 60°C and 30 s at 72°C; and a final extension of 5 min at 72 C. The length and quality of PCR products were verified during electrophoresis with 1% agarose gel. After positive verification, the DNA products were sequenced by Genomed (Warsaw, Poland). The generated sequences were used to perform Basic Local Alignment Search Tool (BLAST) (2) analysis to determine the nearest phylogenetic neighbours against the National Center for Biotechnology Information GenBank database of validly published prokaryotic names.

**Fig. 1.**
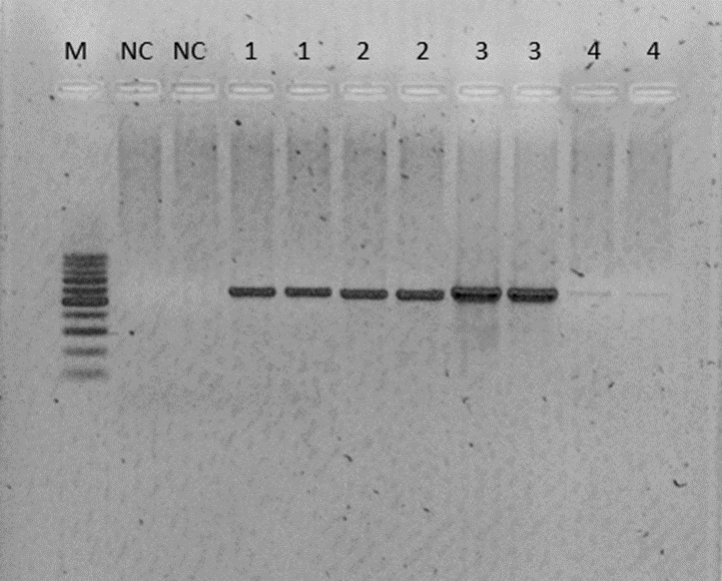
Electrophoretic separation of the PCR products of amplification of one small subunit and three housekeeping genes of bacteria isolated from a dog with periodontitis 1 – *16 srRNA* gene product; 2 – *gyr B1* gene product; 3 – *rpo D1* gene product; 4 – *rpo B1* gene product; M – 100 bp weight marker; NC – negative control

## Results

The bacteria isolated from blood sheep agar were dominated by Gram-negative bacilli with features typical of *P. aeruginosa* (haemolysis, a specific odour and greenish colour), these comprising nearly 99% of all the isolates. However, the colour of the colonies on the Mueller–Hinton agar plates was metallic yellow, which is rarely observed in *P. aeruginosa* cultures. As a result, Gram-negative bacilli were identified based on cultural and morphological characteristics and their growth on MacConkey agar (Oxoid, Basingstoke, UK), on indole production and by oxidase test (bioMérieux, Marcy-l’Étoile, France). Only *P. aeruginosa* grew on MacConkey agar.

The isolate was tested against five classes of antimicrobial agents. In the aminoglycoside class, the strain was sensitive to gentamicin. Resistance was observed to the antipseudomonal cephalosporins (ceftazidime and cefepime), the antipseudomonal carbapenem (imipenem) and the antipseudomonal penicillin combined with β-lactamase inhibitor (piperacillin-tazobactam).

Based on the definition published in Magiorakos *et al*. (21) of multi-drug resistance (MDR) as acquired nonsusceptibility to at least one agent in three or more antimicrobial categories, this strain was classified as MDR. The obtained results prompted the start of treatment with gentamicin at a dose of 4 mg/kg body weight for 10 d, and some improvement was observed after 4 d.

*Pseudomonas aeruginosa* was indicated as a result of biochemical identification as well as identification using the MALDI Biotyper. In the VITEK2 system, the probability was 96%, which corresponds to excellent identification. The MALDI Biotyper scored its identification at 2.28, where score values higher than 1.99 indicated secure to highly probable species identification, values between 1.7 and 1.99 showed probable genus identification and those between 0.0 and 1.69 designated unreliable identification.

The results from BLAST showed that all the gene fragments used in the comparison analysis had the highest similarity to the *P. aeruginosa* sequences deposited in the GenBank database. The query sequence coverage in our analysis ranged between 97 and 100%, and the percentage of identical base pairs ranged from 91% for the *rpoB* gene fragment to 99.81% for the *rpoD* sequence (Table 2). The results of molecular identification confirm that the analysed bacterial isolate was *P. aeruginosa*.

**Table 2.** Results of BLAST used in bacterial isolate molecular identification

Sequence	Query coverage	Percent identity	Length	Taxon	Accession No.
*rpoB*	99%	91%	6,375,262	*Pseudomonas aeruginosa*	CP046602.1
*rpoD*	97%	99.81%	6,375,262	*Pseudomonas aeruginosa*	CP046602.1
*gyrB*	99%	99.6%	6,375,262	*Pseudomonas aeruginosa*	CP046602.1
*16srRNA*	100%	99.42%	1,490	*Pseudomonas aeruginosa*	PP112146.1

## Discussion

Periodontal diseases, including periodontitis, are a significant health issue in dogs that occur in approximately 80% of dogs older than 2 years (10, 29). These diseases not only contribute to discomfort and pain but can also lead to more severe systemic conditions. Additionally, untreated periodontal disease can lead to kidney, liver, lung and heart disease; osteoporosis and diabetes (2). The incidence of the disease increases significantly with the age and weight of the animals. Epidemiological studies indicate a higher occurrence in small breeds (25).

One of the primary aetiological factors for periodontal disease in dogs is bacteria. Pathogenic effects of bacteria result from complex interactions between the microorganisms that inhabit the oral cavity and the host’s immune response. The bacterial flora in the oral cavity of dogs is diverse, containing both Gram-positive and Gram-negative bacteria. *Pseudomonas aeruginosa* is a Gram-negative bacterium known to cause opportunistic infections in humans and animals. It is a highly antibiotic-resistant pathogen that makes the treatment of the infections it causes difficult. In dogs, *P. aeruginosa* can colonise the oral cavity, especially in conditions of weakened immunity or preexisting periodontal diseases, which create a suitable environment for its growth (12). In the presented work we confirmed the presence of *P. aeruginosa* in a dog with periodontitis using different methods: biochemical identification (by VITEK 2), mass spectrometry (with a MALDI Biotyper) and comparison of gene sequences. This confirmation may indicate that *P. aeruginosa* should be considered an aetiological factor of periodontal diseases in dogs.

The pathogenic mechanisms of *P. aeruginosa* in the context of periodontal diseases include the production of tissue-degrading enzymes such as proteases and toxins that can damage host cells (11). Additionally, this bacterium is capable of forming biofilms – complex bacterial structures surrounded by a protective matrix. These biofilms are more difficult to remove through mechanical cleaning and antibiotics (7).

The presence of *P. aeruginosa* in the canine oral microflora is rather unusual (3, 25). It is definitely less common than other bacteria such as *Porphyromonas gulae* or *Porphyromonas gingivalis* (16, 26). However, there are reports showing the occurrence and even dominance of *Pseudomonas* sp. after displacement of *Psychrobacter* as a result of treatment (9). In the study of Riggio *et al*. (25), the bacteria associated with canine periodontal disease and with normal canine oral cavity were identified using 16S rRNA sequencing as well as with a culture-dependent method. Among the results with the culture-dependent method was the finding that 30.9% of the analysed clones were *Pseudomonas* sp. in healthy dogs, but a total 13.9% of isolates were *Pseudomonas* sp. and *P. brenneri* in dogs with periodontitis (25). The results of the studies by Souto *et al*. (27) provided evidence that the oral cavity is a reservoir of *P. aeruginosa*, as well as proof of the association of this microorganism with the presence of periodontal infection in humans. On the other hand, *Pseudomonas* was not identified in a different study concerning canine composite oral microbiota (30). Both our and the other cited results may indicate a yet undiscovered role of *Pseudomonas* in the oral microflora of dogs. The growth of this bacterium in periodontal tissues may reflect its ability to grow rapidly in a competition-depleted environment, which may be reflected in an increase in the abundance of *Pseudomonas* in the intestinal microbiota during disease or after the administration of antimicrobials. Furthermore, the high relative abundance of *Pseudomonas* is unlikely to be due to contamination during periodontitis prevention, because it is difficult to introduce exogenous bacteria and have them proliferate (14, 17).

Treatment and prevention of periodontal diseases in dogs require regular oral hygiene care, including tooth brushing and professional dental cleaning by a veterinarian, and also require an appropriate diet. In cases of bacterial infections, including those caused by *P. aeruginosa*, antibiotics may be necessary, although their use must be strictly controlled because of the increasing resistance of this bacteria (12, 16). For example, one of our results showed the resistance of *P. aeruginosa* to carbapenemase. This finding might be of great importance, because carbapenemase-resistant *P. aeruginosa* strains are amongst the critical pathogens listed on the World Health Organization list of those of high priority (8). In the case presented in this study, 10 days of gentamicin treatment brought significant improvement. On the other hand, in the work of Werckenthin *et al*. (31) *P. aeruginosa* isolates from infections of the skin, ear and mouth as well as the urinary and genital tract of dogs and cats were resistant to most antimicrobial agents or susceptible to them only at high minimum inhibitory concentrations of the drugs (31). However, only 27% of isolates were observed to be resistant to gentamicin.

## Conclusion

Periodontal diseases in dogs are a significant health problem, with bacteria playing a crucial role in their aetiology. Understanding the pathogenic mechanisms of these microorganisms and effective methods of diagnosis and treatment are essential for preventing and controlling these diseases (9). In our opinion, attention should be paid to *Pseudomonas aeruginosa* as a possible aetiological factor of periodontal diseases in dogs.

## References

[1] Albuquerque C., Morinha F., Requicha J., Martins T., Dias I., Guedes-Pinto H., Bastos E., Viegas C. (2012). Canine periodontitis: The dog as an important model for periodontal studies. Vet J.

[2] Altschul S.F., Madden T.L., Schäffer A.A., Zhang J., Zhang Z., Miller W., Lipman D.J. (1997). Gapped BLAST and PSIBLAST: a new generation of protein database search programs. Nucleic Acids Res.

[3] Alves M.C.C., Chaves D.S.A., Benevenuto B.R., de Farias B.O., Coelho S.M.O., Ferreira T.P., Perreira G.A., dos Santos G.C.M., Moreira L.O., de Freitas J.P., Cid Y.P. (2020). Chitosan gels for buccal delivery of *Schinus molle* L. essential oil in dogs: characterization and antimicrobial activity *in vitro*. An Acad Bras Cienc.

[4] Barceló I.M., Torrens G., Escobar-Salom M., Jordana-Lluch E., Capó-Bauzá M.M., Ramón-Pallín C., García-Cuaresma D., Fraile-Ribot P.A., Mulet X., Oliver A., Juan C. (2022). Impact of peptidoglycan recycling blockade and expression of horizontally acquired β-lactamases on *Pseudomonas aeruginosa* virulence. Microbiol Spectr.

[5] Bennik M.H.J., Batt C.A., Tortorello M.L. (1999). Encyclopedia of Food Microbiology.

[6] Clinical and Laboratory Standards Institute (2019). M100-ED29: 2019 Performance Standards for Antimicrobial Susceptibility Testing.

[7] Costerton J.W., Stewart P.S., Greenberg E.P. (1999). Bacterial biofilms: a common cause of persistent infections. Science.

[8] Diggle S.P., Whiteley M. (2020). Microbe Profile: *Pseudomonas aeruginosa*: opportunistic pathogen and labrat. Microbiology.

[9] Flancman R., Singh A., Weese J.S. (2018). Evaluation of the impact of dental prophylaxis on the oral microbiota of dogs. PLoS One.

[10] Garanayak N., Das M., Patra R.C., Biswal S., Panda S.K. (2019). Effect of age on dental plaque deposition and its control by ultrasonic scaling, dental hygiene chew, and chlorhexidine (0.2% w/v) in dogs. Vet World.

[11] Gellatly S.L., Hancock R.E.W. (2013). *Pseudomonas* aeruginosa: new insights into pathogenesis and hostdefenses. Pathog Dis.

[12] Greene C.E., Prescott J.F., Greene C.E., Sykes J.E. (2012). Infectious Diseases of the Dog and Cat.

[13] Harvey C.E., Laster L., Shofer F., Miller B. (2008). Scoring the full extent of periodontal disease in the dog: Development of a total mouth periodontal score (TMPS) system. J Vet Dent.

[14] Hentges D.J., Stein A.J., Casey S.W., Que J.U. (1985). Protective role of intestinal flora against infection with *Pseudomonas aeruginosa* in mice: Influence of antibiotics on colonization resistance. Infect Immun.

[15] Høiby N., Ciofu O., Bjarnsholt T. (2010). Pseudomonas aeruginosa biofilms in cystic fibrosis, Future Microbiol.

[16] Holmstrom S.E., Frost P., Eisner E.R. (2013). Veterinary Dental Techniques for the Small Animal Practitioner.

[17] Kerckhoffs A.P.M., Ben-Amor K., Samsom M., van der Rest M.E., de Vogel J., Knol J., Akkermans L.M.A. (2011). Molecular analysis of faecal and duodenal samples reveals significantly higher prevalence and numbers of *Pseudomonas aeruginosa* in irritable bowel syndrome. J Med Microbiol.

[18] King E.O., Ward M.K., Raney D.E. (1954). Two simple media for the demonstration of pyocyanin and fluorescin. J Lab Clin Med.

[19] Kiyaga S., Kyany’a C., Muraya A.W., Smith H.J., Mills E.G., Kibet C., Mboowa G., Musila L. (2022). Genetic diversity, distribution, and genomic characterization of antibiotic resistance and virulence of clinical *Pseudomonas aeruginosa* strains in Kenya. Front Microbiol.

[20] Klockgether J., Cramer N., Wiehlmann L., Davenport C.F., Tümmler B. (2011). *Pseudomonas aeruginosa* genomic structure and diversity. Front Microbiol.

[21] Magiorakos A.P., Srinivasan A., Carey R.B., Carmeli Y., Falagas M.E., Giske C.G., Harbarth S., Hindler J.F., Kahlmeter G., Olsson-Liljequist B., Paterson D.L., Rice L.B., Stelling J., Struelens M.J., Vatopoulos A., Weber J.T., Monnet D.L. (2012). Multidrug-resistant, extensively drug-resistant and pandrug-resistant bacteria: an international expert proposal for interim standard definitions for acquired resistance. Clin Microbiol Infect.

[22] Matthew M., Sykes R.B. (1977). Properties of the beta-lactamase specified by the *Pseudomonas* plasmid RPL11. J Bacteriol.

[23] Niemiec B.A. (2008). Periodontal disease. Top Companion Anim Med.

[24] Planet P.J, Long S.S., Prober C.G., Fischer M., Kimberlin D. (2023). Principles and Practice of Pediatric Infectious Diseases.

[25] Riggio M.P., Lennon A., Taylor D.J., Bennett D. (2011). Molecular identification of bacteria associated with canine periodontal disease. Vet Microbiol.

[26] Sokolowski J.H. (2021). Periodontal disease in dogs – an update. Vet Focus.

[27] Souto R., Silva-Boghossian C.M., Colombo A.P. (2014). Prevalence of *Pseudomonas aeruginosa* and *Acinetobacte*r spp. in subgingival biofilm and saliva of subjects with chronic periodontal infection. Braz J Microbiol.

[28] Spagnolo A.M., Sartini M., Cristina M.L. (2021). *Pseudomonas aeruginosa* in the healthcare facility setting. Rev Res Med Microbiol.

[29] Stella J.L., Bauer A.E., Croney C.C. (2018). A cross-sectional study to estimate prevalence of periodontal disease in a population of dogs (*Canis familiaris*) in commercial breeding facilities in Indiana and Illinois. PLoS One.

[30] Sturgeon A., Stull J.W., Costa M.C., Weese J.S. (2013). Metagenomic analysis of the canine oral cavity as revealed by high-throughput pyrosequencing of the 16S rRNA gene. Vet Microbiol.

[31] Werckenthin C., Alesík E., Grobbel M., Lübke-Becker A., Schwarz S., Wieler L.H., Wallmann J. (2007). Antimicrobial susceptibility of *Pseudomonas aeruginosa* from dogs and cats as well as *Arcanobacterium pyogenes* from cattle and swine as determined in the BfT-GermVet monitoring program 2004–2006. Berl Munch Tierarztl Wochenschr.

